# Climate Change and the Impact of Greenhouse Gasses: CO_2_ and NO, Friends and Foes of Plant Oxidative Stress

**DOI:** 10.3389/fpls.2018.00273

**Published:** 2018-03-01

**Authors:** Raúl Cassia, Macarena Nocioni, Natalia Correa-Aragunde, Lorenzo Lamattina

**Affiliations:** Instituto de Investigaciones Biológicas, Facultad de Ciencias Exactas y Naturales, Universidad Nacional de Mar del Plata-Consejo Nacional de Investigaciones Científicas y Técnicas, Mar del Plata, Argentina

**Keywords:** climate change, greenhouse effect, oxidative stress, nitric oxide, plants

## Abstract

Here, we review information on how plants face redox imbalance caused by climate change, and focus on the role of nitric oxide (NO) in this response. Life on Earth is possible thanks to greenhouse effect. Without it, temperature on Earth’s surface would be around -19°C, instead of the current average of 14°C. Greenhouse effect is produced by greenhouse gasses (GHG) like water vapor, carbon dioxide (CO_2_), methane (CH_4_), nitrous oxides (N_x_O) and ozone (O_3_). GHG have natural and anthropogenic origin. However, increasing GHG provokes extreme climate changes such as floods, droughts and heat, which induce reactive oxygen species (ROS) and oxidative stress in plants. The main sources of ROS in stress conditions are: augmented photorespiration, NADPH oxidase (NOX) activity, β-oxidation of fatty acids and disorders in the electron transport chains of mitochondria and chloroplasts. Plants have developed an antioxidant machinery that includes the activity of ROS detoxifying enzymes [e.g., superoxide dismutase (SOD), ascorbate peroxidase (APX), catalase (CAT), glutathione peroxidase (GPX), and peroxiredoxin (PRX)], as well as antioxidant molecules such as ascorbic acid (ASC) and glutathione (GSH) that are present in almost all subcellular compartments. CO_2_ and NO help to maintain the redox equilibrium. Higher CO_2_ concentrations increase the photosynthesis through the CO_2_-unsaturated Rubisco activity. But Rubisco photorespiration and NOX activities could also augment ROS production. NO regulate the ROS concentration preserving balance among ROS, GSH, GSNO, and ASC. When ROS are in huge concentration, NO induces transcription and activity of SOD, APX, and CAT. However, when ROS are necessary (e.g., for pathogen resistance), NO may inhibit APX, CAT, and NOX activity by the S-nitrosylation of cysteine residues, favoring cell death. NO also regulates GSH concentration in several ways. NO may react with GSH to form GSNO, the NO cell reservoir and main source of S-nitrosylation. GSNO could be decomposed by the GSNO reductase (GSNOR) to GSSG which, in turn, is reduced to GSH by glutathione reductase (GR). GSNOR may be also inhibited by S-nitrosylation and GR activated by NO. In conclusion, NO plays a central role in the tolerance of plants to climate change.

## Introduction

Life on Earth, as it is, relies on the natural atmospheric greenhouse effect. This is the result of a process in which a planet’s atmosphere traps the sun radiation and warms the planet’s surface.

Greenhouse effect occurs in the troposphere (the lower atmosphere layer), where life and weather occur. In the absence of greenhouse effect, the average temperature on Earth’s surface is estimated around -19°C, instead of the current average of 14°C (Le Treut et al., 2007). Greenhouse effect is produced by greenhouse gasses (GHG). GHG are those gaseous constituents of the atmosphere that absorb and emit radiation in the thermal infrared range (IPCC, 2014). Traces of GHG, both natural and anthropogenic, are present in the troposphere. The most abundant GHG in increasing order of importance are: water vapor, carbon dioxide (CO_2_), methane (CH_4_), nitrous oxides (N_x_O) and ozone (O_3_) (Kiehl and Trenberth, 1997). GHG percentages vary daily, seasonally, and annually.

## GHG Contribute Differentially to Greenhouse Effect

### Water Vapor

Water is present in the troposphere both as vapor and clouds. Water vapor was reported by Tyndal in 1861 as the most important gaseous absorber of variations in infrared radiation (cited in Held and Souden, 2000). Further accurate calculation estimate that water vapor and clouds are responsible for 49 and 25%, respectively, of the long wave (thermal) absorption (Schmidt et al., 2010). However, atmospheric lifetime of water vapor is short (days) compared to other GHG as CO_2_ (years) (IPCC, 2014).

Water vapor concentrations are not directly influenced by anthropogenic activity and vary regionally. However, human activity increases global temperatures and water vapor formation indirectly, amplifying the warming in a process known as water vapor feedback (Soden et al., 2005).

### Carbon Dioxide (CO_2_)

Carbon dioxide is responsible for 20% of the thermal absorption (Schmidt et al., 2010).

Natural sources of CO_2_ include organic decomposition, ocean release and respiration. Anthropogenic CO_2_ sources are derived from activities such as cement manufacturing, deforestation, fossil fuels combustion such as coal, oil and natural gas, etc. Surprisingly, 24% of direct CO_2_ emission comes from agriculture, forestry and other land use, and 21% comes from industry (IPCC, 2014).

Atmospheric CO_2_ concentrations climbed up dramatically in the past two centuries, rising from around 270 μmol.mol^-1^ in 1750 to present concentrations higher than 385 μmol.mol^-1^ (Mittler and Blumwald, 2010; IPCC, 2014). Around 50% of cumulative anthropogenic CO_2_ emissions between 1750 and 2010 have taken place since the 1970s (IPCC, 2014). It is calculated that the temperature rise produced by high CO_2_ concentrations, plus the water positive feedback, would increase by 3–5°C the global mean surface temperature in 2100 (IPCC, 2014).

### Methane (CH_4_)

Methane (CH_4_) is the main atmospheric organic trace gas. CH_4_ is the primary component of natural gas, a worldwide fuel source. Significant emissions of CH_4_ result from cattle farming and agriculture, but mainly as a consequence of fossil fuel use. Concentrations of CH_4_ were multiplied by two since the pre-industrial era. The present worldwide-averaged concentration is of 1.8 μmol.mol^-1^ (IPCC, 2014).

Although its concentration represents only 0.5% that of CO_2_, concerns arise regarding a jump in CH_4_ atmospheric release. Indeed, it is 30 times more powerful than CO_2_ as GHG (IPCC, 2014). CH_4_ generates O_3_ (see below), and along with carbon monoxide (CO), contributes to control the amount of OH in the troposphere (Wuebbles and Hayhoe, 2002).

### Nitrous Oxides (NxO)

Nitrous oxide (N_2_O) and nitric oxide (NO) are GHG. During the last century, their global emissions have rised, due mainly to human intervention (IPCC, 2014). The soil emits both N_2_O and NO. N_2_O is a strong GHG, whereas NO contributes indirectly to O_3_ synthesis. As GHG, N_2_O is potentially 300 times stronger than CO_2_. Once in the stratosphere, the former catalyzes the elimination of O_3_ (IPCC, 2014). In the atmosphere, N_2_O concentrations are climbing up due mainly to microbial activity in nitrogen (N)-rich soils related with agricultural and fertilization practices (Hall et al., 2008).

Anthropogenic emissions (from combustion of fossil fuels) and biogenic emissions from soils are the main sources of NO in the atmosphere (Medinets et al., 2015). In the troposphere, NO quickly oxidizes to nitrogen dioxide (NO_2_). NO and NO_2_ (termed as NO_x_) may react with volatile organic compounds (VOCs) and hydroxyl, resulting in organic nitrates and nitric acid, respectively. They access ecosystems through atmospheric deposition that has an impact on the N cycle as a result of acidification or N enrichment (Pilegaard, 2013).

### NO Sources and Chemical Reactions in Plants

Two major pathways for NO production have been described in plants: the reductive and the oxidative pathways. The reductive pathway involves the reduction of nitrite to NO by NR under conditions such as acidic pH, anoxia, or an increase in nitrite levels (Rockel et al., 2002; Meyer et al., 2005). NR-dependent NO formation has been involved in processes such as stomatal closure, root development, germination and immune responses. In plants, nitrite may also be reduced enzymatically by other molybdenum enzymes such as, xanthine oxidase, aldehyde oxidase, and sulfite oxidase, in animals (Chamizo-Ampudia et al., 2016) or via the electron transport system in mitochondria (Gupta and Igamberdiev, 2016).

The oxidative pathway produces NO through the oxidation of organic compounds such as polyamines, hydroxylamine and arginine. In animals, NOS catalyzes arginine oxidation to citrulline and NO. Many efforts were made to find the arginine-dependent NO formation in plants, as well as of plant NOS (Frohlich and Durner, 2011). The identification of NOS in the green alga *Ostreococcus tauri* (Foresi et al., 2010) led to high-throughput bioinformatic analysis in plant genomes. This study shows that NOS homologs were not present in over 1,000 genomes of higher plants analyzed, but only in few photosynthetic microorganisms, such as algae and diatoms (Di Dato et al., 2015; Kumar et al., 2015; Jeandroz et al., 2016). In summary, although an arginine-dependent NO production is found in higher plants, the specific enzyme/s involved in the oxidative pathways remain elusive.

### Ozone (O_3_)

Ozone (O_3_) is mainly found in the stratosphere, but a little amount is generated in the troposphere. Stratospheric ozone (namely the ozone layer) is formed naturally by chemical reactions involving solar ultraviolet (UV) radiation and O_2_. Solar UV radiation breaks one O_2_ molecule, producing two oxygen atoms (2 O). Then, each of these highly reactive atoms combines with O_2_ to produce an (O_3_) molecule. Almost 99% of the Sun’s medium-frequency UV light (from about 200 to 315 nm wavelength) is absorbed by the (O_3_) layer. Otherwise, they could damage exposed life forms near the Earth surface^1^.

The majority of tropospheric O_3_ appears when NOx, CO and VOCs, react in the presence of sunlight. However, it was reported that NOx may scavenge O_3_ in urban areas (Gregg et al., 2003). This dual interaction between NOx and O_3_ is influenced by light, season, temperature and VOC concentration (Jhun et al., 2015).

Besides, the oxidation of CH_4_ by OH in the troposphere gives way to formaldehyde (CH_2_O), CO, and O_3_, in the presence of high amounts of NOx^1^.

Tropospheric O_3_ is harmful to both plants and animals (including humans). O_3_ affects plants in several ways. Stomata are the cells, mostly on the underside of the plant leaves, that allow CO_2_ and water to diffuse into the tissue. High concentrations of O_3_ cause plants to close their stomata (McAdam et al., 2017), slowing down photosynthesis and plant growth. O_3_ may also provoke strong oxidative stress, damaging plant cells (Vainonen and Kangasjärvi, 2015).

## Global Climate Change: an Integrative Balance of the Impact on Plants

Anthropogenic activity alters global climate by interfering with the flows of energy through changes in atmospheric gasses composition, more than the actual generation of heat due to energy usage (Karl and Trenberth, 2003). Short-term consequences of GHG increase in plants are mainly associated with the rise in atmospheric CO_2_. Plants respond directly to elevated CO_2_ increasing net photosynthesis, and decreasing stomatal opening (Long et al., 2004). To a lesser extent, O_3_ uptake by plants may reduce photosynthesis and induce oxidative stress. In the middle and long term, prognostic consensus about climate change signal a rise in CO_2_ concentration and temperature on the Earth’s surface, unexpected variations in rainfall, and more recurrent and intense weather conditions, e.g., heat waves, drought and flooding events (Mittler and Blumwald, 2010; IPCC, 2014). These brief episodes bring plants beyond their capacity of adaptation; decreasing crop and tree yield (Ciais et al., 2005; Zinta et al., 2014).

Here we will not discuss plants capacity of adaptation to novel environmental conditions when considering large scales and long-term periods. Ecosystems are being affected by climate change at all levels (terrestrial, freshwater, and marine), and it was already reported that species are under evolutionary adaptation to human-caused climate change (for a review see Scheffers et al., 2016). Migration and plasticity are two biological mechanisms to cope with these changes. Data indicate that each population of a species has limited tolerance to sharp climate variations, and they could migrate to find more favorable environments. Habitat fragmentation limits plant movement, being other big threat for adaptation (Stockwell et al., 2003; Leimu et al., 2010). Despite the fact that individual plants are immobile, plant populations move when seeds are dispersed, resulting in differences in the general distribution of the species (Corlett and Westcott, 2013). In this sense, anthropogenic activities also contribute to seed dispersal.

Plasticity is a characteristic related to phenology and phenotype. Phenology is the timing of phases occurrence in the life cycle, and phenotypic plasticity is the range of phenotypes that a single genotype may express depending on its environment (Nicotra et al., 2010). Plasticity is adaptive when the phenotype changes occur in a direction favored by selection in the new environment.

## Climate Change and ROS

Reactive Oxygen Species (ROS) are continuously generated by plants under normal conditions. However, they are increased in response to different abiotic stresses. One of the most important effects of climate change-related stresses at the molecular level is the increase of ROS inside the cells (Farnese et al., 2016). Among ROS, the most studied are superoxide anion ($O_{2}^{•–}$), H_2_O_2_ and the hydroxyl radical (⋅OH^-^).

Reactive Oxygen Species cause damage to proteins, lipids and DNA, affecting cell integrity, morphology, physiology, and, consequently, the growth of plants (Frohnmeyer and Staiger, 2003). The main sources of ROS in stress conditions are: augmented photorespiration, NADPH oxidase (NOX) activity, β-oxidation of fatty acids and disorders in the electron transport chains of mitochondrias and chloroplasts (Apel and Hirt, 2004; AbdElgawad et al., 2015). Hence, higher plants have evolved in the presence of ROS and have acquired pathways to protect themselves from its toxicity. Plant antioxidant system (AS) includes the activity of ROS detoxifying enzymes [e.g., superoxide dismutase (SOD), ascorbate peroxidase (APX), catalase (CAT), glutathione peroxidase (GPX), and peroxiredoxin (PRX)], as well as antioxidant molecules such as ascorbic acid (ASC) and glutathione (GSH) that are present in almost all subcellular compartments (reviewed by Choudhury et al., 2017).

**FIGURE 1:**
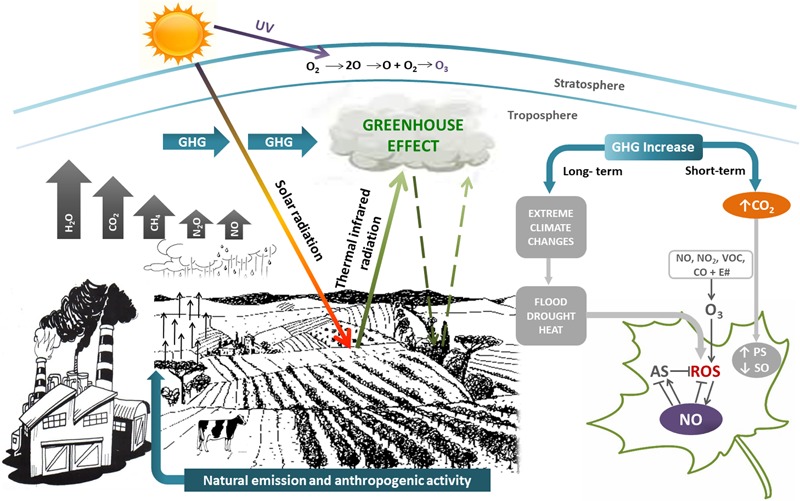
Simplified scheme showing greenhouse gasses (GHG) and their effects on plants. GHG (H_2_O vapor, clouds, CO_2_, CH_4_, N_2_O, and NO) have both natural and anthropogenic origin, contributing to greenhouse effect. Short-term effects of GHG increase is mainly CO_2_ rise, that activates photosynthesis (PS) and inhibits stomatal opening (SO). Long-term effects of GHG increase are extreme climate changes such as floods, droughts, heat. All of them induce the generation of reactive oxygen species (ROS) and oxidative stress in plants. Nitric oxide (NO) could alleviate oxidative stress by scavenging ROS and/or regulating the antioxidant system (AS). GHG and volatile organic compounds (VOC) react in presence of sunlight (E#) to give tropospheric O_3_. Although tropospheric O_3_ is prejudicial for life, stratospheric O_3_ is beneficial, because filters harmful UV-B radiation. The size of arrows are representative of the GHG concentration.

In this context, plants have also developed a tight interaction between ROS and NO as a mechanism to reduce the deleterious consequences of these ROS-induced oxidative injuries. NO orchestrates a wide range of mechanisms leading to the preservation of redox homeostasis in plants. Consequently, NO at low concentration is considered a broad-spectrum anti-stress molecule (Lamattina et al., 2003; Tossi et al., 2009; Correa-Aragunde et al., 2015). **Figure 1** shows the relationship among the different GHG and their impact on plants.

## CO_2_ and NO Contribute to Regulate Redox Homeostasis in Plants

### CO_2_ Increasing: Advantages and Disadvantages

Increased CO_2_ was suggested to have a “fertilization” effect, because crops would increase their photosynthesis and stomatal conductance in response to elevated CO_2_. This belief was supported by studies performed in greenhouses, laboratory controlled-environment chambers, and transparent field chambers, where emitted CO_2_ may be held back and readily controlled (Drake et al., 1997; Markelz et al., 2014). However, more realistic results, obtained by Free-Air Concentration Enrichment (FACE) technology, suggest that the fertilization response due to CO_2_ increase is probably dependent on genetic and environmental factors, and the duration of the study (Smith and Dukes, 2013). An extensive review of the literature in this field made by Xu et al. (2015) concluded that augmented CO_2_ normally increases photosynthesis in C3 species such as rice, soybean and wheat. On the other hand, they pointed out that a negative feedback of photosynthesis could take place in augmented CO_2_, as a result of overload of chemical and reactive generated substrates, leading to an imbalance in the sink:source carbon ratio. Moreover, the energetic cost of carbohydrate exportation increases in elevated CO_2_ level.

The most important photosynthetic enzyme is the ribulose-1,5-bisphosphate carboxylase-oxygenase (RuBisCO). Rubisco is located in mesophyll cells of C3 plants, in direct contact with the intercellular air space linked to the atmosphere by epidermal stomatal pores. Photosynthesis increases at high CO_2_, because Rubisco is not CO_2_ saturated and CO_2_ inhibits the oxygenation reactions and photorespiration (Long et al., 2006). However, long-term high concentration of CO_2_ may down regulate Rubisco activity because ribulose-1,5-bisphosphate is not regenerated. Hexokinase (HXK), a sensor of extreme photosynthate, may participate in the down regulation of Rubisco concentration (Xu et al., 2015). Moreover, severe abiotic stresses, such as temperature and drought, may restrain Rubisco carboxylation and foster oxygenation (Xu et al., 2015).

In C4 crops, such as maize and sorghum, the elevated concentration of CO_2_ inside the bundle sheath cells could prevent a large increase of Rubisco activity at higher atmospheric CO_2_ and, thereby, photosynthetic activity is not augmented. However, at high CO_2_ levels, the water status of C4 plants under drought conditions is improved, increasing photosynthesis and biomass accumulation (Long et al., 2006; Mittler and Blumwald, 2010). That envisages potential advantages for the C4 species in future climatic change scenarios, particularly in arid and semiarid areas.

In addition, high CO_2_ has the benefit of reducing stomatal conductance, decreasing 10% evapotranspiration in both C3 and C4 plants. Simultaneously, the cooling decreased resulting from reduced transpiration causes elevated canopy temperatures of around 0.7°C for most crops. Biomass and yield rise due to high CO_2_ in all C3 plants, but not in C4 plants exception made when water is a restraint. Yields of C3 grain crops jump around 19% on average at high CO_2_ (Kimball, 2016).

Some reports analyze the contribution of CO_2_ in the responses of plants to the combination of multiple stresses. For *Arabidopsis thaliana*, the combination of heat and drought induces photosynthesis inhibition of 62% under ambient CO_2_, but the drop in photosynthesis is just 40% at high CO_2_. Moreover, the protein oxidation increases significantly during a heat wave and drought, and this effect is repressed by increased CO_2_. Photorespiration is also reduced by high CO_2_ (Zinta et al., 2014).

Studying grasses (*Lolium perenne, Poa pratensis*) and legumes (*Medicago lupulina, Lotus corniculatus*) exposed to drought, high temperature and augmented CO_2_, AbdElgawad et al. (2015) demonstrated that drought suppresses plant growth, photosynthesis and stomatal conductance, and promotes in all species the synthesis of osmolytes and antioxidants. Instead, oxidative damage is more markedly observed in legumes than in grasses. In general, warming amplifies drought consequences. In contrast, augmented CO_2_ diminishes stress impact. Reduction in photosynthesis and chlorophyll, as a result of drought and elevated temperature, were avoided by high CO_2_ in the grasses. Noxious effects of oxidative stress, i.e., lipid peroxidation, are phased down in all species by augmented CO_2_. Normally, a reduced impact of oxidative stress is due to decreased photorespiration and diminished NOX activity. In legumes, a rise in levels of antioxidant molecules (flavonoids and tocopherols) contribute as well to the stress mitigation caused by augmented CO_2_. The authors draw the conclusion that these different responses point at an unequal future impact of climate change on the production of agricultural-scale legumes and grass crops.

Kumari et al. (2015) assessed the impact of various levels of CO_2_, ambient (382 ppm) and augmented (570 ppm), and O_3_, ambient (50 ppb) and augmented (70 ppb) on the potato physiological and biochemical responses (*Solanum tuberosum*). They observed that augmented CO_2_ cut down O_3_ uptake, enhanced carbon assimilation, and curbed oxidative stress. Elevated CO_2_ also mitigated the noxious effect of high O_3_ on photosynthesis.

Although some molecular mechanisms underpinning CO_2_ actions are unknown, the results presented highlight the importance of CO_2_ as a regulator that mitigates the potential climate change-induced deleterious consequences in plants. Recent reports suggest that some CO_2_-associated responses may be mediated by NO.

Du et al. (2016) determined that 800 μmol.mol^-1^ of CO_2_ increased the NO concentration in Arabidopsis leaves, through a mechanism related to nitrate availability. Moreover, NO increase, as a consequence of high CO_2_ levels, was reported as a general procedure to improve iron (Fe) nutrition in response to Fe deficiency in tomato roots (Jin et al., 2009).

The gas exchange between the atmosphere and plants is mainly regulated by stomata. But structure and physiology of stomata are also influenced by gasses (García-Mata and Lamattina, 2013). Elevated CO_2_ regulate stomatal density and conductance. Moreover, there is increasing evidence that this response is modified by interaction of CO_2_ with other environmental factors (Xu et al., 2016; Yan et al., 2017). Wang et al. (2015) reported that 800 μmol.mol^-1^ of CO_2_ increases the NO concentration in *A. thaliana* guard cells, inducing stomatal closure. Both NR and NO synthase (NOS)-like activities are necessary for CO_2_-induced NO accumulation. Comprehensive pharmacological and genetic results obtained in Arabidopsis by Chater et al. (2015), show that when CO_2_ concentration is around 700–1000 ppm, stomatal density and closure are reduced. They also illustrate that those elements necessary for this process are: activation of both ABA biosynthesis genes and the PYR/RCAR ABA receptor, and ROS increase. However, Shi et al. (2015) provide genetic and pharmacological evidence that high CO_2_ concentration induces stomatal closure by an ABA-independent mechanism in tomato. They show that 800 μmol.mol^-1^ of CO_2_ increase the expression of the protein kinase OPEN STOMATA 1 (OST1), NOX, and nitrate reductase (NR) genes. They also show that the sequential production of NOX-dependent H_2_O_2_ and NR-produced NO are mainly dependent of OST1, and are involved in the CO_2_-induced stomatal closure.

In ABA-dependent mechanisms, ABA is increased by CO_2._ The binding of ABA to its receptor (PYR/RCAR) inactivates PP2C, activating OST1. In ABA-independent mechanism, OST1 will be transcriptionally induced by CO_2_. Once activated, OST1 along with Ca_2_^+^, activates NOX, increasing ROS (Kim et al., 2010). The rise of guard cells ROS enhances NO, cytosolic free Ca_2_^+^, and pH (Song et al., 2014; Xie et al., 2014). ROS and NO release Ca_2_^+^ from internal reservoirs, or influx external Ca_2_^+^ through plasma membrane Ca_2_^+^_in_ channels. Cytosolic free Ca_2_^+^ inactivate inward K^+^ channels (K^+^_in_) to prevent K^+^ uptake and activate outward K^+^ channels (K^+^_out_) and Cl^-^ (anion) channels (Cl^-^) at the plasma membrane (Blatt, 2000; García-Mata et al., 2003). Ca_2_^+^ also activates slow anion channel homolog 3 (SLAH3), slow anion channel-associated 1 (SLAC1) and aluminum activated malate transporters (ALMT) (Roelfsema et al., 2012). The consequence of the regulation of cation/anion channels is the net efflux of K^+^/Cl^-^/malate and influx of Ca_2_^+^, making guard cells lose turgor by water outlet, causing stomatal closure.

**FIGURE 2:**
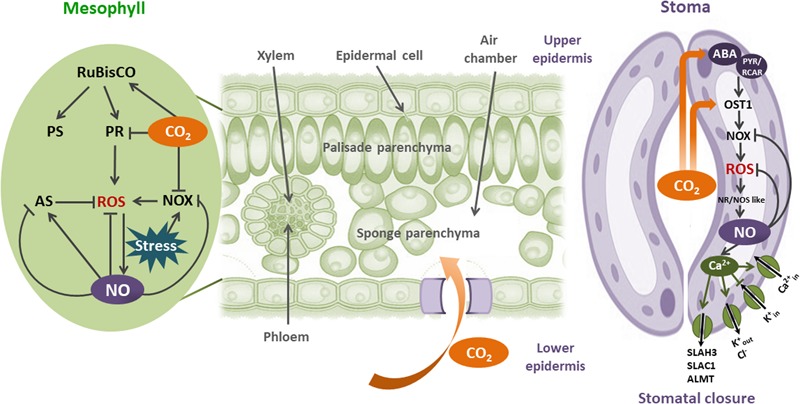
Interplay between CO_2_ and NO in plant redox physiology: CO_2_ enters to the leaves by stomata. Once in mesophyll cells, CO_2_ increase photosynthesis (PS) through the CO_2_-unsaturated Rubisco activity. When plants are in stress environments, ROS could be augmented by Rubisco-induced photorespiration and NADPH oxidase (NOX) activities. NOX- induced $O_{2}^{•–}$, in the apoplast is immediately transformed to H_2_O_2_ by the superoxide dismutase (SOD). Plasma membrane is permeable to H_2_O_2_. CO_2_ moderates oxidative stress in mesophyll cells by inhibiting both Rubisco photorespiration (PR) and NOX activities. Besides, NO is induced by CO_2_ and ROS, alleviating the consequences of oxidative stress by scavenging ROS and activating or inhibiting the antioxidant system (AS). In guard cells, CO_2_ increases the expression and activity of OPEN STOMATA 1 (OST1), in both ABA-dependent and independent mechanisms. OST1 activates NOX, producing ROS and consequently NO increase by nitrate reductase (NR), and NOS-like activities. NO prevents ROS increase by direct scavenging, and inhibiting NOX. NO-dependent Ca_2_^+^ regulated ion channels induces stomatal closure, modulating O_3_ and CO_2_ uptake, decreasing evapotranspiration, and rising leaf temperature.

All together, the results discussed here suggest that CO_2_-induced NO increase is a common plant physiological response to oxidative stresses. **Figure 2** shows the importance of CO_2_ and NO in these processes.

### Abiotic Stress, ROS Generation, and Redox Balance: The Key Role of NO

Reactive oxygen species are generated in apoplast, plasma membrane, chloroplasts, mitochondria, and peroxisomes (Farnese et al., 2016). It was proposed that each stress produces its own “ROS signature” (Choudhury et al., 2017). For instance, drought may reduce the activity of Rubisco, decreasing CO_2_ fixation and NADP+ regeneration by the Calvin cycle. As a consequence, chloroplast electron transport is altered, generating ROS by electron leakage to O_2_ (Carvalho, 2008). In drought stress, ROS increase is produced by NOX activity (Farnese et al., 2016). In flooding, ROS generation is an ethylene-promoted process that involves calcium (Ca^2+^) flux, and NOX activity (Voesenek and Bailey-Serres, 2015).

In heat stress, a NOX-dependent transient ROS rise is an early event (Königshofer et al., 2008). Then, endogenous ROS are sensed through histidine kinases, and an Arabidopsis heat stress factor (HsfA4a) appears to sense exogenous ROS. As a result, the MAPK signal pathway is activated (Qu et al., 2013). Moreover, functional decrease in photosynthetic light reaction induces ROS concentration by high electron leakage from the thylakoid membrane (Hasanuzzaman et al., 2013). In this process, O_2_ is the acceptor, generating $O_{2}^{•–}$.

Thus, individual stresses or their different combinations may produce particular “ROS signatures.” Besides their deleterious effects, ROS are recognized as a signal in the plant reaction to biotic and abiotic stressors. ROS may induce programed cell death (PCD) to avoid pathogen spread (Mur et al., 2008), trigger a systemic defense response signal (Dubiella et al., 2013), or avoid the chloroplast antenna overloading by electrons divert (Choudhury et al., 2017).

Whatever the origin and function, ROS concentration must be adequately regulated to avoid excessive concentration and consequent cellular damages. Depending on NO and ROS concentrations, NO has the dual capacity to activate or inhibit the ROS production, and is a key molecule for keeping cellular redox homeostasis under control (Beligni and Lamattina, 1999a; Correa-Aragunde et al., 2015). NO has a direct ROS-scavenging activity because it holds an unpaired electron, reaching elevated reactivity with O_2_, $O_{2}^{•–}$, and redox active metals. NO can mitigate OH formation by scavenging either Fe or $O_{2}^{•–}$ (Lamattina et al., 2003). However, NO reacting with ROS (mainly $O_{2}^{•–}$) may generate reactive nitrogen species (RNS). An excess of RNS originates a nitrosative stress (Corpas et al., 2011). To avoid the toxicity of nitrosative stress, NO is stored as GSNO in the cell.

#### GSH as a Redox Buffer. GSNO as NO Reservoir. SNO and S-Nitrosylation

Glutathione (GSH) is a small peptide with the sequence γ-l-glutamyl-l-cysteinyl-glycine that has a cell redox homeostatic impact in most plant tissues. It is a soluble small thiol considered a non-enzymatic antioxidant. It exists in the reduced (GSH) or oxidized state (GSSG), in which two GSH molecules are joined by a disulfide bond (Rouhier et al., 2008). GSH alleviates oxidative damages in plants generated by abiotic stresses, including salinity, drought, higher, low temperature, and heavy metals. GSH is precursor of phytochelatins, polymers that chelate toxic metals and transport them to the vacuole (Grill et al., 1989). Studies shown that GSH contributes to tolerate nickel, cadmium, zinc, mercury, aluminum and arsenate heavy metals in plants (Asgher et al., 2017). Moreover, GSH has a role in the detoxification of ROS both directly, interacting with them, or indirectly, participating of enzymatic pathways. GSH is involved in glutathionylation, a posttranslational modification that causes a mixed disulfide bond between a Cys residue and GSH.

GSH can be oxidized to GSSG by H_2_O_2_ and can react with NO to form the nitrosoglutathione (GSNO) derivative. GSNO is an intracellular NO reservoir. It is also a vehicle of NO throughout the cell and organs, spreading NO biological function. GSNO is the largest low-molecular-mass S-nitrosothiol (SNO) in plant cells (Corpas et al., 2013). GSNO metabolism and its reaction with other molecules involve S-nitrosylation and S-transnitrosation which consist of the binding of a NO molecule to a cysteine residue in proteins. Thioredoxin produces protein denitrosylation (Correa-Aragunde et al., 2013). GSNO could be decomposed by the GSNO reductase (GSNOR) to GSSG which, in turn, is reduced to GSH by glutathione reductase (GR).

Glutathione also participates in the GSH/ASC cycle, a series of enzymatic reactions that degrade H_2_O_2_. APX degrades H_2_O_2_ using ASC, the other major antioxidant in plants, as cofactor. The oxidized ASC is reduced by monodehydroascorbate reductase (MDHAR) in an NAD(P)H-dependent manner and by dehydroascorbate reductase (DHAR) employing GSH as electron donor. The resulting GSSG is reduced in turn to GSH by GR (Foyer and Noctor, 2011).

#### Different Effects of NO in the Regulation of Antioxidant Enzymes

The application of NO donors alleviates oxidative stress in plants challenged to abiotic and/or biotic stresses (Laxalt et al., 1997; Beligni and Lamattina, 1999b, 2002; Shi et al., 2007; Xue et al., 2007; Leitner et al., 2009).

Besides the direct ROS-scavenging activity of NO, its beneficial effect is exerted by the regulation of the antioxidant enzymes activity that controls toxic levels of ROS and RNS (Uchida et al., 2002; Shi et al., 2005; Song et al., 2006; Romero-Puertas et al., 2007; Bai et al., 2011). NO can modulate cell redox balance in plants through the regulation of gene expression, posttranslational modification or by its binding to the heme prosthetic group of some antioxidant enzymes.

SOD catalyzes the dismutation of stress-generated $O_{2}^{•–}$ in one of two less harmful species: either molecular oxygen (O_2_) or hydrogen peroxide (H_2_O_2_). APX and CAT are the most important enzymes degrading H_2_O_2_ in plants. They transform H_2_O_2_ to H_2_O and O_2_. APX isoforms are primarily found in the cytosol and chloroplasts, while the CAT isoforms are found in peroxisomes. APX has strong affinity for H_2_O_2_ and uses ASC as an electron donor. In contrast, CAT removes H_2_O_2_ generated in the peroxisomal respiratory pathway without the need to reduce power. Even though CAT affinity for H_2_O_2_ is low, its elevated rate of reaction offers an effective way to detoxify H_2_O_2_ inside the cell. PRX may reduce both hydroperoxide and peroxynitrite.

Many reports on different plant species demonstrate that NO induces the transcription and activity of antioxidative enzymes in response to oxidative stress. The tolerance to drought and salt-induced oxidative stress in tobacco is related to the ABA-triggered production of H_2_O_2_ and NO. In turn, they induce transcripts and activities of SOD, CAT, APX, and GR (Zhang et al., 2009). UV-B-produced oxidative stress in *Glycine max* was alleviated by NO donors, which induced transcription and activities of SOD, CAT, and APX (Santa-Cruz et al., 2014). Furthermore, in bean leaves, SOD, CAT, and APX activities are increased by NO donors, and protected from the oxidative stress generated by UV-B irradiation (Shi et al., 2005). Drought tolerance in bermudagrass is improved by ABA-dependent SOD and CAT activities. This effect is regulated by H_2_O_2_ and NO, NO acting downstream H_2_O_2_ (Lu et al., 2009).

Several antioxidant enzymes have been identified as target of S-nitrosylation, resulting in a change of their biological activity (Romero-Puertas et al., 2008; Bai et al., 2011; Fares et al., 2011). For instance, NO reinforces recalcitrant seed desiccation tolerance in *Antiaris toxicaria* by activating the ascorbate-glutathione cycle through S-nitrosylation to control H_2_O_2_ accumulation. Desiccation treatment reduced the level of S-nitrosylated APX, GR, and DHAR proteins. Instead, NO gas exposure activated them by S-nitrosylation (Bai et al., 2011). Furthermore, APX was S-nitrosylated at Cys32 during saline stress and biotic stress, enhancing its enzymatic activity (Begara-Morales et al., 2014; Yang et al., 2015). In addition, auxin-induced denitrosylation of cytosolic APX provoked inhibition of its activity, followed by an increase of H_2_O_2_ concentration and the consequent lateral root formation in Arabidopsis (Correa-Aragunde et al., 2013). Moreover, an inhibitory impact of S-nitrosylation on APX activity was also reported during programmed cell death in Arabidopsis (de Pinto et al., 2013). CAT was identified to be S-nitrosylated in a proteomic study of isolated peroxisomes (Ortega-Galisteo et al., 2012). A decrease of S-nitrosylated CAT under Cd treatment was reported. In addition, *in vitro* experiments demonstrated a reversible inhibitory effect of APX and CAT activities by NO binding to the Fe of the heme cofactor (Brown, 1995; Clark et al., 2000). In addition, NOXs have been involved in plant defense, development, hormone biosynthesis and signaling (Marino et al., 2012). Whereas S-nitrosylation did not affect SOD activities, nitration inhibited Mn-SOD1, Fe-SOD3, and CuZn-SOD3 activity to different degrees (Holzmeister et al., 2015). SOD isoforms could also regulate endogenous NO availability by competing for the common substrate, $O_{2}^{•–}$, and it was demonstrated that bovine SOD may release NO from GSNO (Singh et al., 1999). When GSNO is decomposed by GSNOR, it produces GSSG. GSNOR is also regulated by NO. Frungillo et al. (2014) demonstrated that NO-derived from nitrate assimilation in Arabidopsis inhibited GSNOR1 by S-nitrosylation, preventing GSNO degradation. They proposed that (S)NO controls its own generation and scavenging by modulating nitrate assimilation and GSNOR1 activity. It was also shown that chilling treatment in poplar increased S-nitrosylation of NR, along with a significant decrease of its activity (Cheng et al., 2015).

The dual activity of Prx, suggests a role for this enzyme both in ROS and RNS regulation. S-nitrosylation of Arabidopsis PrxIIE inhibits its peroxynitrite activity, increasing peroxynitrite-mediated tyrosine nitration (Romero-Puertas et al., 2007). Pea mitochondrial PrxIIF was S-nitrosylated under salt stress, and its peroxidase activity was reduced by 5 mM GSNO (Camejo et al., 2013).

An interesting study demonstrated that NO controls hypersensitive response (HR) through S-nitrosylation of NOX, inhibiting ROS synthesis. This triggers a feedback loop limiting HR (Yun et al., 2011).

Other proteins related to abiotic stress response are regulated by S-nitrosylation (For a review see Fancy et al., 2017).

**FIGURE 3:**
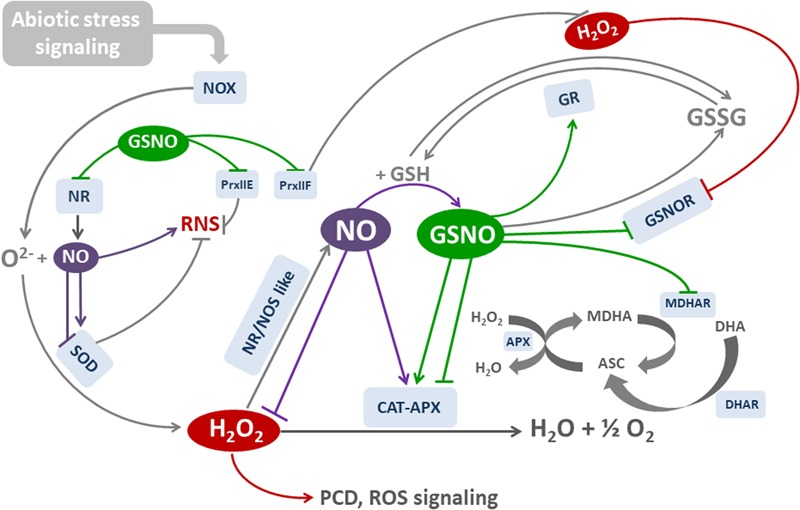
Molecules and mechanisms involved in NO-mediated redox balance. H_2_O_2_ is generated mainly by NOX and SOD as a response to (a)biotic stress. APX and CAT are the main H_2_O_2_-degrading enzymes. NO is increased by H_2_O_2_ through the induction of NR/NOS-like activities, and may scavenge ROS or induce both the transcription and activity of SOD, CAT, and APX. In parallel, NO is combined with GSH to form nitrosoglutathione GSNO. GSNO regulates many enzymatic activities by the posttranslational modification of cysteine residues through S-Nitrosylation. NOX and CAT activities are inhibited by S-nitrosylation, whereas APX is either activated or inhibited by S-nitrosylation. NO also inhibits APX by binding to heme group. GSNO is degraded by GSNOR, which could be inhibited by H_2_O_2_ and S-nitrosylation.NR could be inhibited by S-nitrosylation. GR reduces GSSG to GSH, and it is activated by S-nitrosylation. Ascorbate (ASC) is a cofactor of APX. Reduced ASC is generated by MDHAR and DHAR, using GSH as electron donor. Both enzymes are inhibited by S-nitrosylation. Reactive Nitrogen Species (RNS) may be originated by NO and $O_{2}^{•–}$ reaction. SOD regulate RNS dismutating $O_{2}^{•–}$. Peroxiredoxins (Prx) reduce both ROS AND RNS. RNS are degraded by PrxIIe, and H_2_O_2_ by PrxIIF. Both enzymes are inhibited by S-nitrosylation. Red lines: H_2_O_2_-regulated reactions. Purple lines: NO-regulated reactions. Green lines: GSNO-regulated reactions.

**Figure 3** is a simplified diagram that illustrates the main oxidative and nitrosative effects that modulate the activities of key cell components, thus maintaining cell redox balance. Note the feedback and positive-negative regulatory processes occurring in the main pathways. They involve posttranslational modifications that activate and inhibit the components involved in cell antioxidant system.

## Conclusions and Perspectives

The accelerating rate of climate change, together with habitat fragmentation caused by human activity, are part of the selective pressures building a new Earth’s landscape.

Climate change is a multidimensional and simultaneous variation in duration, frequency and intensity of parameters like temperature and precipitation, altering the seasons and life on the Earth. In this scenario, plant species with increased adaptive plasticity will be better equipped to tolerate changes in the frequency of extreme weather events. GHG are one of the forces driving climate change. However, CO_2_ and NO may contribute to maintaining the cell redox homeostasis, regulating the amount of ROS, GSH, GSNO, and SNO.

In this manuscript, we summarize the available evidence supporting the presence of broad spectrum anti-stress molecules, as NO in plants, for coping with unprecedented changes in environmental conditions. Future research should focus in better understanding the influence of GHG on plant physiology.

## Author Contributions

RC conceived the project and wrote the manuscript. MN drew figures and collaborated in writing the manuscript. NC-A and LL supervised and complemented the drafting. All the persons entitled to authorship have been named and have approved the final version of the submitted manuscript.

## Conflict of Interest Statement

The authors declare that the research was conducted in the absence of any commercial or financial relationships that could be construed as a potential conflict of interest. The reviewer MCR-P and handling Editor declared their shared affiliation.

## Footnotes

1 https://ozonewatch.gsfc.nasa.gov/facts/ozone.html
